# Penetrating renal injuries: an observational study of non-operative management and the impact of opening Gerota’s fascia

**DOI:** 10.1186/s13017-022-00439-7

**Published:** 2022-06-20

**Authors:** Thomas W. Clements, Chad G. Ball, Andrew J. Nicol, Sorin Edu, Andrew W. Kirkpatrick, Pradeep Navsaria

**Affiliations:** 1 Cumming School of Medicine, University of Calgary, Calgary, Canada; 2 Groote Schuur Hospital, University of Cape Town, Main Road, Observatory, Cape Town, 7925 South Africa

**Keywords:** Penetrating solid organ injury, Renal trauma, Non-operative management

## Abstract

**Background:**

Non-operative management has become increasingly popular in the treatment of renal trauma. While data are robust in blunt mechanisms, the role of non-operative management in penetrating trauma is less clear. Additionally, there is a paucity of data comparing gunshot and stab wounds.

**Methods:**

A retrospective review of patients admitted to a high-volume level 1 trauma center (Groote Schuur Hospital, Cape Town) with penetrating abdominal trauma was performed. Patients with renal injuries were identified and compared based on mechanism [gunshot (GSW) vs. stab] and management strategy (operative vs. non-operative). Primary outcomes of interest were mortality and failure of non-operative management. Secondary outcomes of interest were nephrectomy rates, Clavien-Dindo complication rate, hospital length of stay, and overall morbidity rate.

**Results:**

A total of 150 patients with renal injuries were identified (82 GSW, 68 stab). Overall, 55.2% of patients required emergent/urgent laparotomy. GSWs were more likely to cause grade V injury and concurrent intra-abdominal injuries (*p* > 0.05). The success rate of non-operative management was 91.6% (89.9% GSW, 92.8% stab, *p* = 0.64). The absence of hematuria on point of care testing demonstrated a negative predictive value of 98.4% (95% CI 96.8–99.2%). All but 1 patient who failed non-operative management had associated intra-abdominal injuries requiring surgical intervention. Opening of Gerota’s fascia resulted in nephrectomy in 55.6% of cases. There were no statistically significant risk factors for failure of non-operative management identified on univariate logistic regression.

**Conclusions:**

NOM of penetrating renal injuries can be safely and effectively instituted in both gunshot and stab wounds with a very low number of patients progressing to laparotomy. Most patients fail NOM for associated injuries. During laparotomy, the opening of Gerota’s fascia may lead to increased risk of nephrectomy. Ongoing study with larger populations is required to develop effective predictive models of patients who will fail NOM.

## Background

Penetrating abdominal trauma has undergone a massive shift in recent decades. The historical experience of mandatory laparotomies has been supplanted by selective non-operative management (NOM) based on clinical risk factors and advanced diagnostic imaging [1–7]. While blunt renal trauma has long been managed conservatively with fantastic results, conservative management of penetrating trauma to the kidney has only more recently been explored [8–13]. The candidacy for non-operative management, as well as the risk factors for failure, remain poorly defined [7, 12, 13]. Additionally, differences in penetrating mechanisms (gunshot vs. stab), have yet to be studied extensively.

Large, multi-centered experience has shown that grade of injury and concomitant abdominal injury are risk factors for failure of non-operative management in penetrating renal trauma [8, 10]. Once in the operating theater, the risk that these patients undergo radical nephrectomy is not insignificant [14]. Increased understanding of the patient population who may be managed without intervention represents an opportunity to preserve patient renal function, decreases dialysis rates, and minimizes the overall clinical burden of traumatic renal injuries. The clinical course of patient undergoing NOM, especially those sustaining gunshot wounds (GSW), is becoming increasingly well-defined [15–18]. However, an in depth understanding of the differences in patients who fail, and more specifically why they fail, is elusive.

While blunt renal injuries predominate the North American trauma literature, penetrating trauma happens with greater incidence in many low- and middle-income countries [12]. In these settings, cross-sectional imaging is less common place and the clinical exam is paramount. This manuscript reports the largest single center experience with penetrating renal trauma to date. This retrospective data serves to further delineate patients who may or may not require operative management of penetrating renal injuries. Additionally, a comparison between stab and gunshot wounds is made.

## Methods

### Database design

The Groote Schuur Hospital (GSH) in Cape Town, South Africa, is a high-volume level 1 trauma center which prospectively maintains a database of all patients presenting with penetrating abdominal trauma. All patients are included regardless of age or ISS. Patients who die in the trauma bay prior to admission to the ward are not included. Patients sustaining major thoracic, cardiac, or neurosurgical trauma are also excluded. Database metrics include presenting demographics, comorbidities, vitals, injury severity, diagnostic testing, and procedures/interventions. Outcomes recorded include length of hospital/ICU stay, complications, disposition, and mortality data.

## Patient population

All patients presenting to GSH with isolated, penetrating, abdominal trauma from April 30, 2015, to January 30th, 2019, were retrospectively reviewed. Patients diagnosed with any grade of renal trauma through operative or computed tomography (CT) findings were included. All patients suffering stab or gunshot wounds were included. Patients sustaining combined penetrating and blunt mechanisms were excluded. Patients were analyzed as a whole, as well as in stab versus gunshot wound subgroups. Patients who did not undergo immediate, urgent laparotomy after their initial assessment were classified as having undergone “Non-operative Management” (NOM). Patients undergoing delayed laparotomy after admission for NOM were analyzed in an intention to treat fashion as part of the NOM group. Patients taken urgently to the operating theater on admission for any reason were deemed “Operative Management” (OM). All patients were included regardless of injury severity or outcomes. Morbidity was graded based on the Clavien-Dindo classification system. Patient files were reviewed by two independent reviewers.

### Analysis

Primary outcomes of interest included failure of NOM, mortality, and complications rates. Secondary outcomes included hospital LOS, ICU LOS, and rates of renal failure. All statistics were done using commercially available SPSS® software. Categorical variables were analyzed using Chi-squared, or Fischer’s exact test where appropriate. Means of continuous variables were compared using the students T test. Medians were compared using Mann–Whitney U test. Univariate logistic regressions were employed to identify factors contributing to failure of NOM. Standard statistical methodology was employed (*p* < 0.05 = significant).

## Results

A total of 899 patients were admitted with penetrating abdominal trauma (GSW = 563, Stab = 336). One hundred and fifty patients were diagnosed with a penetrating renal injury (GSW = 54.7%, Stab = 45.3%). Isolated renal trauma was encountered in 50 patients (33.3%). Concurrently injured organs were most commonly the liver (40.7%), diaphragm (25.3%), and colon (24.0%). No resuscitative thoracotomies were performed in either group. Patients sustaining GSW’s were more severely injured, more often presented with peritonitis, had a higher rate of concurrent injury, and more often had unstable vitals (*p* < 0.05). Demographic and vitals information is given in Table 1.

**Table 1:** Presenting clinical and demographic data from patients admitted to GSH with penetrating renal injuries from April 30, 2015 to January 30, 2019

	Overall (*N* = 150)	GSW (*N* = 82)	Stab (*N* = 68)	*p*-value
Median age (year) (IQR)	25.5 (IQR = 21–31)	26.5 (IQR = 21–31.5)	25.0 (IQR = 22–31)	0.56
% Male	95.3%	93.9%	97.1%	0.36
Median # of wounds	2 (IQR = 1–4)	2 (IQR = 1–4)	2 (IQR = 1–4)	0.56
HIV status (% positive)	5.3%	7.3%	2.9%	0.23
Mean presenting HR (bpm ± 95% CI)	91.4 ± 3.7	95.6 ± 5.2	86.2 ± 3.2	0.01
Mean presenting SBP (mmHg ± 95% CI	128.6 ± 3.9	129.0 ± 6.0	128.2 ± 4.7	0.41
Mean ISS	21.3 ± 2.7	28.2 ± 4.2	13.2 ± 1.7	< 0.05
Presenting pH	7.33 ± 0.02	7.32 ± 0.03	7.33 ± 0.02	0.30
Initial lactate	2.8 ± 0.5	3.1 ± 0.6	2.5 ± 0.9	0.26
Presenting Hb	126.2 ± 2.0	130.0 ± 2.6	115.0 ± 1.3	0.78
Peritonitis (%)	36.7	58.5	10.2	< 0.05
Hemodynamic Instability (%)	6.0	8.5	2.9	0.15
Isolated renal injury (%)	33.3	6.1	66.2	< 0.05

*Y* years, *IQR* Interquartile range, *SBP* systolic blood pressure, *ISS* injury severity score, *Hb* hemoglobin, *GSW* Gunshot wound. Hemodynamic instability is defined as a SBP < 90 mmHG, or HR > 100 beats per minute

### Initial investigations

Of all patients (*n* = 899) admitted with penetrating abdominal trauma, 110 (12.2%, 95%CI 10.2–14.6) were reported to have gross hematuria (stab = 26.4% (95%CI 18.4–35.6%), GSW = 73.6% (95%CI 64.4–81.6%)), 42.7% (95%CI 33.3–52.5%)) of which did not have a renal injury. Microhematuria was observed in 332 patients (Stab = 38.6% (95%CI 33.3–44.0%), GSW = 58.4% (95%CI 52.9–63.8%)), 244 (73.5%) of which did not have a renal injury. Overall, any form of hematuria had a 95.3% sensitivity (95% CI 90.5–98.1%) and 60.3% (95%CI 56.7–63.9%) specificity for renal injury, with a negative predictive value of 98.4% (95% CI 96.8–99.2%). Of patients with kidney injuries who required immediate laparotomy, 94.0% (95%CI 90.5–98.1%) had hematuria, 44.7% (95%CI 36.3–53.3%) of which was gross hematuria. The negative predictive value of clear urine (no gross or microscopic hematuria) for ruling out a renal injury in a patient requiring immediate laparotomy for any reason was 98.7% (95%CI 96.3–99.6%).

Of all patients, 496 (55.2%) underwent urgent laparotomy. Immediate laparotomy based on clinical exam alone was performed in 351 patients (70.5%). An immediate laparotomy following admission CT scan was performed in 145 patients (34.0%). Urgent CT scan at the time of admission was deemed appropriate in 426 patients (47.4%).

Of patients with a renal injury (*n* = 150), 73.3% had an urgent CT scan. Renal injury was diagnosed at the time of emergent laparotomy without CT scan in 40 patients (26.7%). The most common indication for laparotomy in patients with renal injuries was peritonitis (67.8%), followed by radiographic findings at CT scan (18.5%), and hemodynamic instability (12.3%). Of patients with renal injuries taken for emergent laparotomy, 84.6% had sustained gunshot wounds.

AAST grading of renal injuries found on CT scan or intraoperatively are given in Table 2. The most common injuries were grade III (39.3%), and Grade IV (30.0%). Gross hematuria was not associated with higher AAST grade kidney injuries (Grade IV/V) (*p* = 0.22).

**Table 2:** American association for the surgery of trauma (AAST) grade of renal injuries in patients with penetrating stab and gunshot wounds (GSW) admitted to GSH during the study period

AAST grade	Stab (*n* = 68)	GSW (*n* = 82)	*p*-value
I	2	3	1.0
II	16	11	0.15
III	26	33	0.06
IV	24	21	0.19
V	0	14	0.0001

### Operative management

**Fig. 1:**
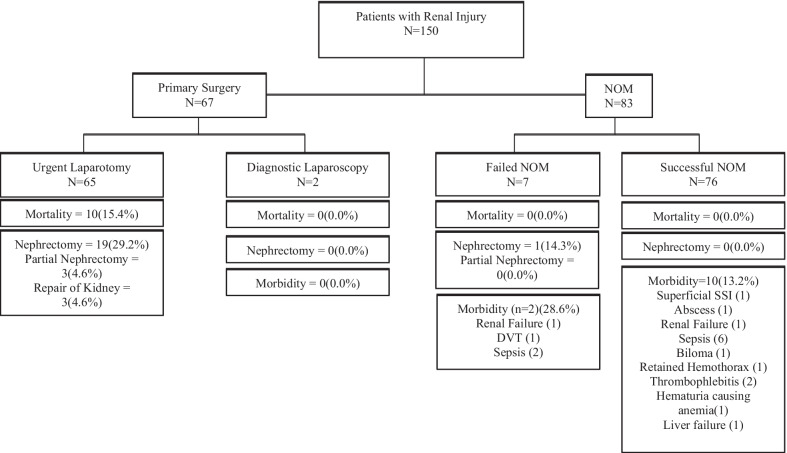
Clinical decision pathways and outcomes for all patients with renal injuries. NOM = Non-operative management. Failure of NOM was defined as a need for any abdominal surgical intervention. Successful NOM patients were managed without the need for surgical intervention. All patients in diagnostic laparoscopy group were planned, delayed operations for left sided thoracoabdominal stab wounds. DVT = Deep Vein Thrombosis

**Fig. 2:**
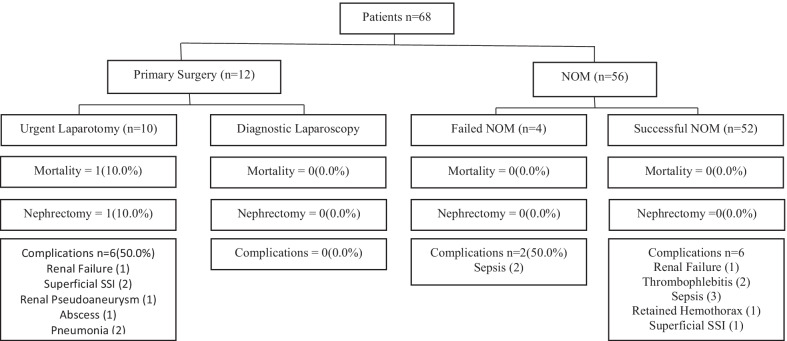
Clinical decision pathways and outcomes for patients sustaining abdominal stab wounds causing renal injuries. NOM = Non-operative management. Failure of NOM was defined as a need for laparotomy. Successful NOM patients were managed without the need for surgical intervention. Primary surgery is defined as the need for emergent/urgent laparotomy directly from the emergency department prior to admission to a hospital ward/intensive care unit. SSI = Surgical Site Infection

**Fig. 3:**
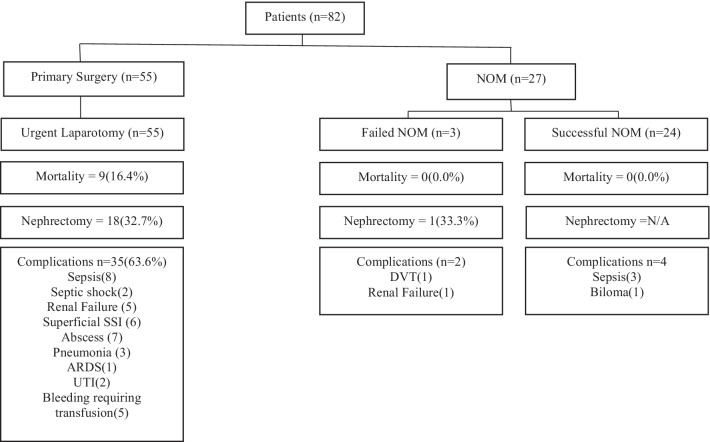
Clinical decision pathways and outcomes for patients sustaining abdominal gunshot wounds causing renal injuries. NOM = Non-operative management. Failure of NOM was defined as a need for laparotomy. Successful NOM patients were managed without the need for surgical intervention. Primary surgery is defined as the need for emergent/urgent laparotomy directly from the emergency department prior to admission to a hospital ward/intensive care unit

Management decisions and outcomes are shown in Figs. 1, 2 and 3. Overall, 55.3% of all patients with kidney injuries were managed non-operatively. Non-operative management was successful in 91.6% of cases. Patients with gunshot wounds were taken to the OR much more commonly than patients with stab wounds (67.1% vs. 14.7%) (*p*-value < 0.001). Gunshot wounds were much more likely to cause AAST grade V injuries (*p* = 0.0001), which in turn were far more likely to be managed operatively compared to all other grades (92.8% vs. 39.7%, *p* < 0.001). In patients with isolated renal trauma (*n* = 50), only four (8.0%) required laparotomy, all for open kidney repair, none of which required nephrectomy. In all patients going to the OR who underwent total or partial nephrectomy, 100.0% required additional surgical intervention. Of these, 95.7% required major visceral repair/resection, or damage control surgery. The remaining patient required simple liver packing for an AAST Grade II liver injury and was closed primarily. Patients with isolated renal trauma were caused by stab wounds 90% of the time. Patients with gunshot wounds to the abdomen that cause renal injuries were far more likely to have concurrent intra-abdominal injuries (93.9% vs. 33.8%, *p* < 0.01). The overall rate of nephrectomy for all patients (OM and NOM) was 13.3% (95%CI 8.3–19.8%). Nephrectomy was far more common in GSW than stab wounds (23.1% vs. 1.5%, *p*-value < 0.0001). Other interventions included partial nephrectomy (2.0%), primary repair (2.0%), and “other” procedures (exploration only, hemorrhage control with electrocautery, and simple packing) (6.7%). For patients in whom Gerota’s fascia was opened, total nephrectomy was the most common procedure at a rate of 55.6% (95%CI 38.7–72.3%, *p* < 0.001), partial nephrectomy occurred in 8.3% (95%CI 0.0–17.7%), open repair in 8.3% (95%CI 0.0–17.7%), and “other” procedures (packing, simple exploration, application of energy devices/hemostatics) in 27.8% (95%CI 12.6–42.9%). Patients undergoing total nephrectomy (20) were more likely to have grade IV (8, 40%), or V (12, 60%) injuries (*p* < 0.001). All patients undergoing partial nephrectomy (3) had AAST grade IV injuries. Primary repair was performed in one patient with a grade IV injury, and two patients with grade III injuries. Hemorrhage control, exploration, or simple packing were performed in three grade IV, three grade III, three grade II, and one grade I injury.

### Non-operative management

Non-operative management was successful in 91.6% of cases. Rates of success of NOM were not different between GSW and stab groups (89.9% vs. 92.8%, *p*-value = 0.64). Univariate logistic regression for factors predicting failure of non-operative management is given in Table 3. None of the recorded variables were reliable predictors of NOM failure on univariate analysis, and therefore multivariate analysis was not performed. In patients undergoing NOM, five (6.0%) required angioembolization (four kidney, one liver). A total of four (4.8%) patients required percutaneous drainage of collections, all due to abscess or biloma from concurrent liver injuries. A single patient is represented with frank hematuria after discharge with no findings on repeat CT angiogram and spontaneous resolution. No patient required cystoscopy or nephrostomy. Of the seven (8.4%) patients failing NOM, only one required nephrectomy. This patient additionally required damage control for deterioration of a severe liver injury. A single patient underwent an exploration of the kidney only. The remainder failed due to hollow viscous injury (2), hemorrhage from the spleen (1), wound sepsis requiring debridement (1), and concern for ongoing liver hemorrhage (1).

**Table 3:** Univariate logistic regression for factors predictive of failure in NOM

Characteristic	Univariate logistic regression *p*-value
Age	1.0
GSW	0.55
AAST grade	
I	1.0
II	0.532
III	0.774
IV	0.714
V	–
High-grade injury (III–V)	0.55
SBP < 90	1.0
HR > 100	0.53
ISS	0.94
Lactate	0.87
Gross hematuria	0.65
pH	0.11

*SBP* Systolic Blood Pressure, *HR* Heart Rate, *ISS* injury severity score, *AAST* American Association for the Surgery of Trauma, *GSW* Gunshot Wound. High-grade injuries were defined as AAST grade III, IV, and V injuries

Comparisons of NOM and OM are given in Table 4. Compared to patients undergoing operative management, patients undergoing NOM had lower mortality rates, hospital length of stay, and complication rates, but were also less severely injured. In patients who were management non-operatively, there were no differences in patients sustaining GSW versus stab wounds with regard to mortality (0.0 vs. 0.0%, *p* = 1.0), overall complication rate (18.5% vs. 12.5%, *p*-value = 0.53), Clavien-Dindo III/IV complication rate (11.1% vs. 7.1%, *p*-value = 0.54), or readmission rate (7.4% vs. 8.9%, *p*-value = 0.90). The median length of stay was longer in GSW victims managed non-operatively versus stab victims (5.0 vs. 3.0, *p* = 0.018). Comparisons of GSW and stab outcomes are given in Table 5.

**Table 4:** Comparisons of outcomes in non-operative management (NOM) and operative management (OM) for patients with penetrating renal trauma

Outcome measure	Overall (*n* = 150)	NOM (*n* = 83)	OM (*n* = 67)	*p*-value
Mortality	7.3%	1.2%	14.9%	0.001
Median length of stay (days)	5.0	4.0	8.5	< 0.001
Mean ICU LOS (days)	4.1	6.0	3.9	0.14
Mean ventilated days (days)	3.2	2.0	3.3	0.29
Overall complication rate	35.3%	14.4%	61.2%	< 0.001
Clavien-Dindo III/IV complication rate	19.3%	8.4%	32.8%	< 0.001
Readmission rate	9.3%	8.4%	10.4%	0.67

*LOS* Length of Stay

**Table 5:** Subgroup analysis of stab wounds and gunshot wounds managed by non-operative management

Outcome measure	Stab (*n* = 68)			GSW (*n* = 82)			NOM of Stab versus GSW *p*-value
	OM (12)	NOM (56)	*p*-value	OM (55)	NOM (27)	*p*-value	
Mortality (%)	8.3	0.0	0.17	16.4	0.0	0.026	1.0
Median LOS (days)	5.0	3.0	0.045	9.0	5.0	0.046	0.018
Mean ICU LOS (days)	3.0	1.0	–	4.0	12.0	–	–
Mean Ventilated Days (days)	2.0	0.0	–	3.4	2	–	–
Overall complication (%)	50.0	12.5	0.003	63.6	18.5	0.0001	0.53
Clavien-Dindo III/IV (%)	25.0	7.1	0.10	34.5	11.1	0.033	0.54
Readmission Rate (%)	16.7	8.9	0.60	9.1	7.4	0.80	0.90

*LOS* Length of stay, *GSW* Gunshot wound, *OM* Operative management, *NOM* Non-operative management

## Discussion

Much work has been done in the last two decades to delineate trauma populations benefiting from restraint on the part of the trauma surgeon [5, 8, 9]. Advancements in percutaneous therapies have also revolutionized the management of solid organ injuries. As a retroperitoneal structure, the kidney is theoretically a prime candidate for NOM. The body’s ability to tamponade the retroperitoneum, along with the ease of embolization and/or minimally invasive urologic interventions would predict that a number of kidney injuries can be managed without immediate surgical intervention. In contrast to blunt trauma in which parenchymal injury dominates, missile trajectories in penetrating trauma seemingly could introduce a higher likelihood of injury to the larger vascular and calyceal structure [5–8, 10]. Perhaps unsurprisingly, the NOM of penetrating renal injuries has therefore lagged behind. Furthermore, most studies have grouped stab and gunshot wounds into a single group [8, 10, 11, 13, 14]. Differences in the outcomes in NOM of gunshot versus stab wounds are relatively sparse. Delineating the mechanism and frequency of failure of NOM allows for improved management of the pathology.

Overall, NOM was safe and effective with appropriate selection. With a mortality rate of 0.0% and a success rate of 91.6%, the safety profile of NOM was excellent. Patients requiring immediate laparotomies almost always require operation due to associated injuries, regardless of the grade of renal insult. This is echoed in NOM, where the majority of failures are due to concomitant injuries. Only one patient who failed NOM actually required a nephrectomy. If requirement for renal intervention only is taken as the criteria for failure, NOM is successful in 97.6% of cases. Furthermore, NOM was safe in both gunshot and stab wounds with appropriate selection, with no statistical difference in outcomes or failure rates between the two mechanisms. While fewer patients are candidates, the NOM of renal gunshot wounds has an equivalent success rate to stab wounds.

Patients were rarely taken to the operating room for intervention on the kidney alone. In both up-front OM, and failed NOM, indications for laparotomy were almost always present independent of the renal injury. Regardless, if Gerota’s fascia was opened intraoperatively, more than half of these patients had their kidney removed. This study and many others show that even high-grade injuries can be managed non-operatively, regardless of grade [1, 3, 5]. Additionally, there were patients in this dataset with grade V renal injuries who underwent laparotomy in whom renal salvage was attained by not violating Gerota’s fascia. While there are many clinical scenario’s which require expeditious nephrectomy, the exploration of Gerota’s fascia alone dramatically increases the chances of renal loss. The decision to explore the kidney should therefore be made thoughtfully in light of the overwhelming success of NOM.

The most pressing question in NOM is patient selection. At GSH, the standard indications of peritonitis, hemodynamic instability, and bowel evisceration are used to select patients for up-front operative management. Examinable patients lacking the above indications will then undergo CT scanning based on clinical exam, hematuria results, and missile tract. Patients meeting the above criteria can almost always be reliably treated non-operatively. Patients without hematuria do not routinely undergo CT scanning in the absence of a concerning missile tract or physical exam. The concept that NOM is safe may be helpful in setting where resources may be scarce, or unreliably available.

This study has multiple limitations. The retrospective nature of the study creates the possibility of reporting bias. Additionally, the granular aspects of surgical management of the injured kidney are difficult to elucidate, as are the reasons for failure of NOM in cases of multiple abdominal or missed injuries. The power of the study is likely a limitation in the determination of significant predictors for failure. AAST grade and acidosis have been previously identified as risk factors for nephrectomy [8, 10], neither of which was borne out in this study. As these outcomes are rare in NOM, the study was likely not powered to detect these risk factors.

These data demonstrate that NOM can be equally effective in gunshot and stab wounds when patient selection is appropriate. Nephrectomy is a rare occurrence, and complication rates are relatively low. Additionally, patients undergoing NOM most often fail due to associated injuries to other abdominal organs, as opposed to the kidney itself. The effective selection of patients for NOM has the potential to decrease unnecessary nephrectomies, decreases resource utilization, and overall improves patient outcomes.

## Conclusion

NOM of penetrating renal injuries can be safely and effectively instituted in both gunshot and stab wounds with a very low number of patients progressing to laparotomy. Most patients fail NOM for associated injuries. During laparotomy, the opening of Gerota’s fascia may lead to decompression of controlled hemorrhage, and subsequent risk of nephrectomy. Ongoing study with larger population is required to develop effective predictive models of patients who will fail NOM.

## Data Availability

The datasets generated and/or analyzed during the current study are not publicly available due to patient privacy restrictions, but are available from the corresponding author on reasonable request.

## Abbreviations

AAST: American association for the surgery of trauma
CT: Computed tomography
GSH: Groote Schuur Hospital
GSW: Gunshot wound
ICU: Intensive care unit
ISS: Injury severity score
LOS: Length of stay
NOM: Non-operative management
OM: Operative management
